# Nematode eel parasite found inside acanthocephalan cysts – a “Trojan horse” strategy?

**DOI:** 10.1186/s13071-014-0504-8

**Published:** 2014-11-18

**Authors:** Sebastian Emde, Sonja Rueckert, Judith Kochmann, Klaus Knopf, Bernd Sures, Sven Klimpel

**Affiliations:** Goethe-University (GU), Institute for Ecology, Evolution and Diversity; Biodiversity and Climate Research Centre (BiK-F), Senckenberg Gesellschaft für Naturforschung (SGN), Max-von-Laue-Str. 13, D-60438 Frankfurt/ M, Germany; School of Life, Sport and Social Sciences, Edinburgh Napier University, Edinburgh, EH11 4BN UK; Leibniz-Institute of Freshwater Ecology and Inland Fisheries, Ecophysiology and Aquaculture, Müggelseedamm 310, D-132587 Berlin, Germany; Faculty of Biology, Department of Aquatic Ecology, University Duisburg-Essen, Universitätsstr. 5, D-45141 Essen, Germany

**Keywords:** *Anguillicoloides crassus*, Invasive species, *Neogobius melanostomus*, Life cycle strategy, Parasite infection, Intermediate host, Hyperparasitism

## Abstract

**Background:**

The invasive eel parasite *Anguillicoloides crassus* (syn. *Anguillicola crassus*) is considered one of the major causes for the decline of the European eel (*Anguilla anguilla*) panmictic population. It impairs the swim bladder function and reduces swimming performance of its host. The life cycle of this parasite involves different intermediate and paratenic hosts. Despite an efficient immune system of the paratenic fish hosts acting against infections with *A. crassus*, levels of parasitized eels remain high in European river systems. Recently, the round goby *Neogobius melanostomus* (Gobiidae) has become dominant in many rivers in Europe and is still spreading at a rapid pace. This highly invasive species might potentially act as an important, so far neglected paratenic fish host for *A. crassus.*

**Methods:**

Based on own observations and earlier single sightings of *A. crassus* in *N. melanostomus*, 60 fresh individuals of *N. melanostomus* were caught in the Rhine River and examined to assess the infection levels with metazoan parasites, especially *A. crassus*. Glycerin preparations were used for parasite identification.

**Results:**

The parasite most frequently found in *N. melanostomus* was the acanthocephalan *Pomphorhynchus* sp. (subadult stage) which occurred mainly encysted in the mesenteries and liver. Every third gobiid (P = 31.7%) was infected by *A. crassus* larvae (L3) which exclusively occurred inside the acanthocephalan cysts. No intact or degenerated larvae of *A. crassus* were detected elsewhere in the goby, neither in the body cavity and mesenteries nor in other organs. Affected cysts contained the acanthocephalan larvae and 1–12 (mI =3) living *A. crassus* larvae. Additionally, encysted larvae of the nematode *Raphidascaris acus* were detected in the gobies, but only in the body cavity and not inside the acanthocephalan cysts.

**Conclusions:**

Based on our observations, we suggest that *A. crassus* might actively bypass the immune response of *N. melanostomus* by invading the cysts of acanthocephalan parasites of the genus *Pomphorhynchus* using them as “Trojan horses”. Providing that eels prey on the highly abundant round goby and that the latter transfers viable infective larvae of *A. crassus*, the new paratenic host might have a strong impact on the epidemiology of *A. crassus*.

## Background

The European eel (*Anguilla anguilla*) has high economic value since it is considered a culinary delicacy in many European and Asian countries. However, the IUCN Red List of threatened species classifies it as a critically endangered species due to its dramatic decline in recruitment since the early 1980’s. The spawning stock biomass is estimated to range from 2% to 12% of its former size. The recruitment of glass eels has dropped to only 5% of the mean values throughout the distribution area and less than 1% for the North Sea recorded from 1960 to 1979 and recovery is highly unlikely. Since the end of the 1970’s the eel catch size in Europe has thus decreased by more than 75% e.g. [1].

**Figure 1:**
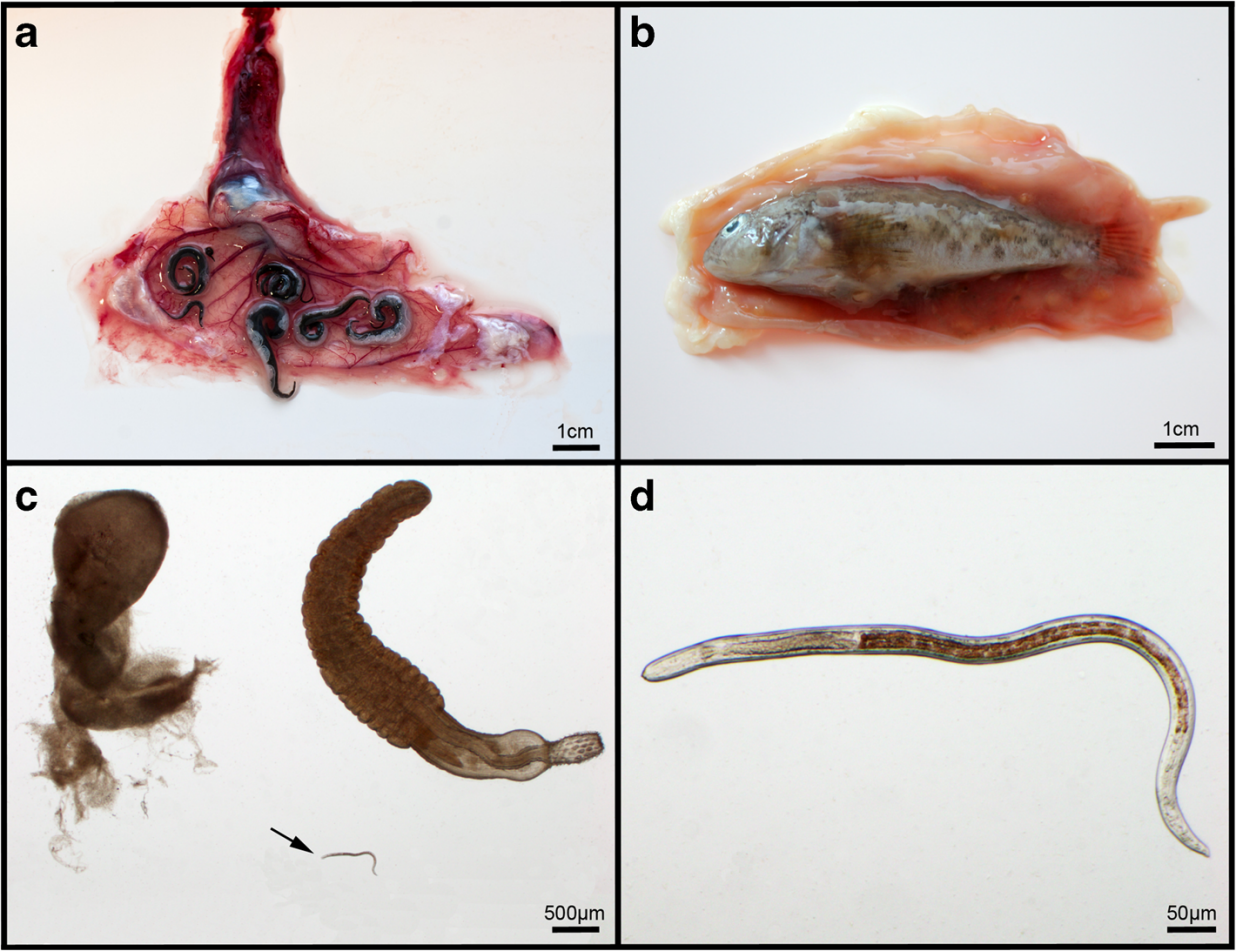
**Hosts and parasites. a** Opened up swim bladder of the European eel *Anguilla anguilla* showing a common number of adult blood sucking *Anguillicoloides crassus*, which can cause heavy infections and might hamper the 5000 km catadromous spawning migration to the Sargasso Sea; **b** The invasive round goby *Neogobius melanostomus* as prey organism in an opened up eel stomach; **c** Opened up acanthocephalan cyst (upper left) of *Pomphorhynchus* sp. (right), a highly abundant parasite of the goby, and third-stage larvae of *A. crassus*, isolated from the cyst (arrow); **d** Higher magnification of the L3 larvae of *A. crassus.*

Strong anthropogenic pressures exerted by fishing, pollutant levels, increasing habitat loss through engineering work on watercourses, as well as so-called turbine losses at hydro-electric power stations are causing many populations to decline [1]. However, biological causes, such as increased predation pressure by fish-eating birds (particularly cormorants), diseases caused by viruses (e.g. *Herpesvirus anguillae*) as well as debilitating anguillicolosis caused by the invasive parasite *Anguillicoloides crassus* (Figure 1a; syn. *Anguillicola crassus*) found in the swim bladder, are also considered as factors significantly contributing to the population decline [2,3]. Infestations with *A. crassus* lead to significant impairment of the swim bladder function [4] and reduced swimming performance [5]. This can have fatal consequences during the 5000 km spawning migration when eels undertake daily vertical migrations between depths of 200 and 1000 m [6].

*A. crassus* has been listed as one of the 100 ’worst’ exotic species in Europe [7] fulfilling the most stringent criteria of invasiveness [8-10], i.e. its human-driven expansion far beyond its native home range, as well as being abundant, well established and critically harmful in its new environment. Probably introduced with eels imported from Taiwan [11,12], *A. crassus* has successfully invaded Europe and the European eel. First records of *A. crassus* in Europe date back to 1982 in North-West Germany [13]. It can now be found in the eel stocks in almost all of Europe [14]. Both, experimental and field studies indicate that the European eel is more susceptible to *A. crassus* than the Japanese eel [15-18]. Obviously, the immune system of the co-evolved natural host, the Japanese eel, is more effective against the larvae of the nematode. The Japanese eel is also capable of eliminating the parasite after vaccination [19] or under high infection pressure [18], but this has not been observed in European eels.

Adult, ovoviviparous *A. crassus* are localized exclusively in the swim bladder of its final host, the eel, where it feeds on blood and reproduces. Embryonated eggs (containing the second larval stage, the first one already developed in the uterus) as well as hatched second-stage larvae (L2) leave the eel via the *ductus pneumaticus* of the swim bladder and the intestinal tract. The nematode uses different invertebrates (especially planktonic crustaceans) as obligate first intermediate hosts for the development of its third-stage larvae (L3), which is infective for the eel (e.g. so far, 23 different crustacean species, mostly copepods could be identified as first intermediate hosts) [20]. Additionally, 50 paratenic hosts such as several insect and amphibian species as well as at least 37 fish species can be incorporated in the life cycle [14,20]. The paratenic hosts accumulate large numbers of parasites, either freely in the body cavity, or in and on organs such as the gonads, intestinal wall and swim bladder [21,22], and thus, bridge the trophic levels between larger piscivorous eels and copepods. Smaller eels get infected predominantly by feeding on parasitized crustacean intermediate hosts, whereas larger eels, preying mostly on fish, ingest infective larvae with paratenic fish hosts. Within paratenic hosts the third-stage larvae of *A. crassus* elicit a cellular immune response which can lead to the encapsulation and killing of the worm [22]. Even larvae that are still viable, but encapsulated, loose their infectivity to the eel [23]. The intensity and efficiency of the host response against *A. crassus* differs greatly between fish species, which is an important factor determining the suitability of a fish species as paratenic host. In general, physoclist fish species appear to be more suitable paratenic hosts than physostome species [21,24].

One of the worst invasive fish species in Europe is the round goby *Neogobius melanostomus*, which originates from the Ponto-Caspian region (Black and Caspian Sea) and now often shares habitats (e.g. river Rhine and Main) with the European eel [25,26]. During a study which focused on trophic interactions and parasite fauna of *N. melanostomus*, cysts of acanthocephalans were isolated from freshly captured fish specimens and kept separately (Emde unpublished). After approx. two hours, individuals of nematodes were accidentally observed outside the acanthocephalan cysts. These were unambiguously identified as third-stage larvae of *A. crassus* by using morphological features according to the descriptions of Moravec [27] (Figure 1d). Main characteristics are 1) the small terminal spike at the tail tip and 2) the rounded cephalic end (mouth), which provides two small lateral, anteriorly directed sclerotized teeth. Both are followed by a sclerotized apparatus at the posterior end, slightly visible as bifurcate in the lateral view (Figure 1d). Based on these first observations of *A. crassus* inside the acanthocephalan cysts as well as knowledge of former single findings of *A. crassus* in *N. melanostomus* [28,29] a sampling of *N. melanostomus* was undertaken in June 2013 at the Rhine River, Hesse, Germany. The aim was to collect quantitative data on prevalence and intensities of *A. crassus* that would allow reliable confirmation of the so far overlooked hyperparasitic behaviour.

## Methods

A total of 60 fresh individuals of *N. melanostomus* were sampled at the Rhine River (49°51′54.7′N 8°21′40.2″E) using a fishing rod. Infection levels with metazoan parasites, especially with *A. crassus,* were assessed using a stereomicroscope. Skin, fins and gills were inspected for ectoparasites. Afterwards, body cavity as well as inner organs like gastrointestinal tract, gonads, kidney, liver, mesenteries, spleen and eyes were dissected and examined for endoparasites. Beside the nematode *Raphidascaris acus*, many acanthocephalan cysts (*Pomphorhynchus* sp.) were found in the liver and mesenteries of the organs in the body cavity and stored separately in the cavities of a multi-well culture plate filled with physiological NaCl solution (0.9%). All acanthocephalan cysts were carefully observed and dissected (outer wall, wall and inside of cyst) to check for the larvae of *A. crassus*. Afterwards, all parasites were stored in 70% ethanol. For parasite identification glycerin preparations were made according to Riemann [30]. A microscope was used to examine and document the parasites. The nematodes were determined with descriptions of Moravec [27]. The parasitological terminology including prevalence (P), intensity (I), mean intensity (mI) and mean abundance (mA) followed Bush et al. [31].

## Results and discussion

The parasite most frequently found in *N. melanostomus* was the acanthocephalan *Pomphorhynchus* sp. (P = 88.3%, mI = 13.5) (Table 1). Subadult *Pomphorhynchus* sp. (Figure 1c), of which 95% were encysted, were located in the mesenteries and liver. These results are consistent with those obtained four years earlier from gobies sampled 262 km downstream [32]. *Neogobius melanostomus* become infected with *Pomphorhynchus* sp. by preying on parasitized amphipods which act as obligate first intermediate hosts. Whether *N. melanostomus* is used as a suitable paratenic host or simply represents a dead end for the life cycle of *Pomphorhynchus* sp. remains unclear [32]. Apart from acanthocephalan cysts, encysted nematode larvae of *Raphidascaris acus* were detected in the body cavity and liver of *N. melanostomus* (P = 36.7%, mI = 3.2) (Table 1). This nematode is a widespread parasite, probably using *N. melanostomus* as intermediate host to finally reach its final host such as pike (*Esox lucius*) and trout (*Salmo trutta*) via predation [32,33]. However, a successful transmission of *Pomphorhynchus* and *Raphidascaris* from *N. melanostomus* to their final hosts remains to be explored.

**Table 1:** **Parasite fauna of** ***Neogobius melanostomus*** **from Rhine River**

**Parasite species**	**Stage**	**Site**	**P [%]**	**I**	**mI**	**mA**
Nematoda						
***Raphidascaris acus***	larv.	BC	36.7	1-12	3.2	1.2
***Anguillicoloides crassus***	larv.	AC	31.7	1-12	3.0	1.0
Acanthocephala						
***Pomphorhynchus*** **sp.**	larv.	L/Mes	88.3	1-69	13.5	12.0

AC = acanthocephalan cyst, BC = body cavity, I = Intensity, larv. = larvae, L = liver, mA = mean abundance, Mes = mesentery, mI = mean intensity and P = prevalence.

*Neogobius melanostomus* has been extensively studied for parasite fauna e.g. [32,34,35] and a few records of the nematode’s larvae in *N. melanostomus,* and *N. kessleri*, another Ponto-Caspian gobiid, exist for the Danube River [28,36]. Due to the low infection levels (P = 2% - 20% and mA = 0.02 - 0.26) they have been interpreted as accidental infections by these authors. Kvach [29] also detected *A. crassus* as a rare parasite of *N. melanostomus* from the Baltic Sea (P = 6.6% and mA = 0.1), where L3 have been found encysted on the internal organs. However, *A. crassus* larvae were never found at high prevalence, neither in the body cavity nor in or on the organs. In this study, every third gobiid (P = 31.7%) was infected by third-stage larvae of *A. crassus* (Table 1) which in turn were infesting some of the acanthocephalan cysts (Figure 1c). Affected cysts contained the acanthocephalan larvae, and in addition 1–12 (mI = 3) living *A. crassus* larvae. This rather high prevalence disproves the assumption that Ponto-Caspian gobies play no significant role in the life cycle of *A. crassus*.

Drastic alterations in biodiversity and faunal composition are currently taking place in the largest European rivers, effectively leading to a species turnover of crustaceans and fish [37,38]. Gobiid fish species such as *N. melanostomus* are effective invaders in Central Europe. They originate from the Ponto-Caspian Basin and have spread very quickly to the Rhine River via the Main-Danube Canal connecting the Danube and Rhine Rivers [32,39]. Today, invasive Ponto-Caspian gobiids share river habitats of the North Sea catchment area with the European eel. Our investigations revealed that *A. crassus* larvae occur abundantly in *N. melanostomus*, which could serve as a paratenic host. Although hitherto there are no quantitative stomach analyses that prove *N. melanostomus* as prey of the European eel, individuals of *N. melanostomus* are commonly found in stomachs of eels >1 kg fresh weight (Franz Schwab professional fisherman, personal communication and own observation) (Figure 1b). Furthermore, both fish species share the same habitat, and the broad and piscivorous diet of the European eel suggests that it will adopt or might have already adopted the highly abundant *N. melanostomus* as an important new prey item.

Although further studies are clearly needed to better understand transmission pathways of *A. crassus*, we believe that the high infection rates including high prevalence and intensity of viable *A. crassus* larvae inside acanthocephalan cysts would enhance chances to reach the definitive host, the eel. Thus, *N. melanostomus* would function as a paratenic host where *A. crassus* effectively avoids the direct consequences of the goby’s immune response for a longer time by utilizing the acanthocephalan cysts as “Trojan horses”. This phenomenon could also be described as ‘facultative hyperparasitism’. Hyperparasitism involving acanthocephalans was found in the cockroach *Periplaneta americana* infected by the acanthocephalan *Moniliformis moniliformis* [40]. The author described that larvae of the cestode *Hymenolepis diminuta* are able to penetrate the acanthocephalan cysts and utilize its protective function in order to develop. It was argued that *P. americana* might show some kind of immune-depression which supports the infectiousness of the cestode that would not survive under normal conditions. Hyperparasitism of eel parasites was described by Freeman and Shinn [41] and Aguilar et al. [42] before; they observed protozoan parasites (myxosporeans) infecting metazoan parasites, such as monogeneans and digeneans. Interestingly, Moravec et al. [43] reported a case in young eels (8–16 cm), with some third-stage larvae of *A. crassus* found inside the cuticle of adult nematodes. This phenomenon was attributed to a very limited space of the swim bladder in the small-sized eels. However, it seems unlikely that nematode larvae observed here ended up inside the acanthocephalan cysts due to restricted space inside the gobies as *A. crassus* larvae were exclusively found inside the cysts of the acanthocephalan and never free or separately encapsulated in the body cavity, mesenteries or other tissue. We therefore suggest that the larvae are moving in a more purposive and directed manner. If this strategy could also be proven for other potentially paratenic fish hosts which are similarly infected with encapsulated *Pomphorhynchus* sp. individuals such as the ruffe (*Gymnocephalus cernua*), it would help to explain the occasionally high *A. crassus* infestation rates in the European eel population.

## Conclusions

This study provides first evidence for a possible new strategy of the larval nematode parasite *A. crassus* to escape the host’s defence by using acanthocephalan cysts as a hiding place. We hope to trigger new research activities into this kind of hyperparasitic behaviour, in order to confirm the infectivity of the larvae (L3) from these cysts and further test our assumptions that *N. melanostomus* represents a good portion of the diet of eels, but also that the immune-evasion strategy of *A. crassus* can also be found in other fish species.

## References

[1] ICES (2012). Report of the joint EIFAAC/ICES working group on Eels (WGEEL). Int Council Expl Sea ICES CM 2012/ACOM.

[2] Van Ginneken V, Ballieux B, Willemze R, Coldenhoff K, Lentjes E, Antonissen E, Haenen O, van den Thillart G (2005). Hematology patterns of migrating European eels and the role of EVEX virus. Comp Biochem Physiol, Part C.

[3] Sures B, Knopf K (2004). Parasites as a threat to freshwater eels?. Science.

[4] Würtz J, Taraschewski H, Pelster B (1996). Changes in gas composition in the swimbladder of the European eel (*Anguilla anguilla*) infected with *Anguillicola crassus* (Nematoda). Parasitology.

[5] Palstra A, Heppener D, Van Ginneken V, Székely C, van den Thillart G (2007). Swimming performance of silver eels is severely impaired by the swim-bladder parasite *Anguillicola crassus*. J Exp Mar Biol Ecol.

[6] Arestrup K, Økland F, Hansen MM, Righton D, Gargan P, Castonguay M, Bernatchez L, Howey P, Sparholt H, Pedersen MI, McKinley RS (2009). Oceanic spawning migration of the European eel (*Anguilla anguilla*). Science.

[7] Minchin D: **Species factsheet -*****Anguillicola crassus*****.** *DAISIE (Delivering Alien Invasive Species Inventories Europe)*, 2008 [http://www.europe-aliens.org/speciesTheWorst.do]

[8] Davis MA, Thompson K (2000). Eight ways to be a colonizer; two ways to be an invader: a proposed nomenclature scheme for invasion biology. Bull Ecol Soc Am.

[9] Colautti RI, MacIsaac HJ (2004). A neutral terminology to define ‘invasive’ species. Diversity Distrib.

[10] ISSG: **IUCN/SSC Invasive Species Specialist Group** [http://www.issg.org] (Accessed Oct. 2013).

[11] Køie M (1991). Swim-bladder nematodes (*Anguillicola* spp.) and the gill monogeneans (*Pseudodactylogyrus* spp.) parasitic on the European eel (*Anguilla anguilla*). J Cons int Explor Mer.

[12] Wielgoss S, Taraschewski H, Meyer A, Wirth T (2008). Population structure of the parasitic nematode *Anguillicola crassus*, an invader of declining North Atlantic eel stocks. Mol Ecol.

[13] Neumann W (1985). Schwimmblasenparasit *Anguillicola* bei Aalen. Fisch Teichwirt.

[14] Kirk RS (2003). The impact of *Anguillicola crassus* on European eels. Fisheries Manag Ecol.

[15] Knopf K, Mahnke M (2004). Differences in susceptibility of the European eel (*Anguilla anguilla*) and the Japanese eel (*Anguilla japonica*) to the swim-bladder nematode *Anguillicola crassus*. Parasitology.

[16] Knopf K (2006). The swimbladder nematode *Anguillicola crassus* in the European eel *Anguilla anguilla* and the Japanese eel *Anguilla japonica*: differences in susceptibility and immunity between a recently colonized host and the original host. J Helminthol.

[17] Münderle M, Taraschewski H, Klar B, Chang CW, Shiao JC, Shen KN, He JT, Lin SH, Tzeng WN (2006). Occurrence of *Anguillicola crassus* (Nematoda: Dracunculoidea) in Japanese eels *Anguilla japonica* from a river and an aquaculture unit in SW Taiwan. Dis Aquat Org.

[18] Heitlinger E, Laetsch D, Weclawski U, Han YS, Taraschewski H (2009). Massive encapsulation of larval *Anguillicoloides crassus* in the intestinal wall of Japanese eels. Parasite Vector.

[19] Knopf K, Lucius R (2008). Vaccination of eels (*Anguilla japonica* and *Anguilla anguilla*) against *Anguillicola crassus* with irradiated L3. Parasitology.

[20] Lefebvre F, Fazio G, Crivelli AJ: ***Anguillicoloides crassus.*** In *Fish parasites: Pathobiology and Protection.* Edited by Woo PTK, Buchmann K. Wallingford, UK: CABI; 2012:310–326.

[21] Székely C (1994). Paratenic hosts for the parasitic nematode *Anguillicols crassus* in Lake Balaton, Hungary. Dis Aquat Org.

[22] Székely C, Pazooki J, Molnár K (1996). Host reaction in paratenic fish hosts against 3rd stage larvae of *Anguillicola crassus*. Dis Aquat Org.

[23] Székely C (1996). Experimental studies on the infectivity of *Anguillicola crassus* third-stage larvae (Nematoda) from paratenic hosts. Folia Parasitol.

[24] Thomas K, Ollevier F (1992). Paratenic hosts of the swimbladder nematode *Anguillicola crassus*. Dis Aquat Org.

[25] Froese R, Pauly D: *FishBase. World Wide Web electronic publication*. Version 8/2014. http://www.fishbase.org

[26] Roche KF, Janač M, Jurajda P (2013). A review of Gobiid expansion along the Danube-Rhine corridor–geopolitical change as a driver for invasion. Knowl Manag Aquat Ec.

[27] Moravec F (2013). Parasitic Nematodes of Freshwater fishes of Europe.

[28] Francová K, Ondračková M, Polačik M, Jurajda P (2011). Parasite fauna of native and non-native populations of *Neogobius melanostomus* (Pallas, 1814) (Gobiidae) in the longitudinal profile of the Danube River. J Appl Ichthyol.

[29] Kvach Y (2004). The far eastern nematode *Anguillicola crassus* - new parasite of the invasive round goby *Neogobius melanostomus* in the Baltic Sea. Vestn Zool.

[30] Riemann F, Higgins RP, Thiel H (1988). Nematoda. Introduction to the Study of Meiofauna.

[31] Bush O, Lafferty AD, Lotz JM, Shostak AW (1997). Parasitology meets ecology on his own terms: Margolis et al. revisited. J Parasitol.

[32] Emde S, Rueckert S, Palm HW, Klimpel S (2012). Invasive Ponto-Caspian amphipods and fish increase the distribution range of the acanthocephalan *Pomphorhynchus tereticollis* in the river Rhine. Plos One.

[33] Ondračová M, Dávidová M, Pečínková M, Blažek R, Gelnar M, Valová Z, Cerný J, Jurajda P (2005). Metazoan parasites of *Neogobius* fishes in the Slovak section of the River Danube. J Appl Ichthyol.

[34] Rakauskas V, Bacevičius E, Pūtys Ž, Ložys L, Arbačiauskas K (2008). Expansion, feeding and parasites of the round goby, *Neogobius melanostomus* (Pallas, 1811), a recent invader in the Curonian Lagoon, Lithuania. Acta Zool Lituanica.

[35] Molnár K (2006). Some remarks on parasitic infections of the invasive *Neogobius* spp. (Pisces) in the Hungarian reaches of the Danube River, with a description of *Goussia szekelyi* sp. n. (Apicomplexa: Eimeriidae). J Appl Ichthyol.

[36] Ondračková M, Dávidová M, Blažek R, Gelnar M, Jurajda P (2009). The interaction between an introduced fish host and local parasite fauna: *Neogobius kessleri* in the middle Danube River. Parasitol Res.

[37] Haas G, Brunke M, Streit B, Leppäkoski E, Gollasch S, Olenin S (2002). Fast Turnover in Dominance of Exotic Species in the Rhine River Determines Biodiversity and Ecosystem Function: An Affair Between Amphipods and Mussels. Invasive Aquatic Species of Europe: Distribution, Impacts, and Management.

[38] Bernauer D, Jansen W (2006). Recent invasions of alien macroinvertebrates and loss of native species in the upper Rhine River, Germany. Aquat Inv.

[39] Panov VE, Alexandrov B, Arbačiauskas K, Binimelis R, Copp GH, Grabowski M, Lucy F, Leuven RSEW, Nehring S, Paunović M, Semenchenko V, Son MO (2009). Assessing the risks of aquatic species invasions via European inland waterways: From concepts to environmental indicators. Integr Environ Assess Manag.

[40] Taraschewski H (2000). Host-parasite interactions in Acanthocephala: a morphological approach. Adv Parasit.

[41] Freeman MA, Shinn AP (2011). Myxosporean hyperparasites of gill monogeneans are basal to the Multivalvulida. Parasite Vector.

[42] Aguilar A, Aragort W, Alvarez MF, Leiro JM, Sanmartín M (2004). Hyperparasitism by *Myxidium giardia* Cépède 1906 (Myxozoa: Myxosporea) in *Pseudodactylogyrus bini* (Kikuchi, 1929) Gussev, 1965 (Monogenea: Dactylogyridae), a parasite of the European eel *Anguilla anguilla* L. Bull Eur Assn Fish P.

[43] Moravec F, Di Cave D, Orecchia P, Paggi L (1994). Experimental observations on the development of *Anguillicola crassus* (Nematoda: Dracunculoidea) in its definitive host, Anguilla anguilla (Pisces). Folia Parasit.

